# Concentration-Dependent Multi-Potentiality of L-Arginine: Antimicrobial Effect, Hydroxyapatite Stability, and MMPs Inhibition

**DOI:** 10.3390/molecules26216605

**Published:** 2021-10-31

**Authors:** Mohammed Nadeem Bijle, Mallikarjuna Rao Pichika, Kit-Kay Mak, Abhishek Parolia, Muneer Gohar Babar, Cynthia Yiu, Umer Daood

**Affiliations:** 1 Paediatric Dentistry, Faculty of Dentistry, The University of Hong Kong, Hong Kong; mnbijle@connect.hku.hk; 2 Pharmaceutical Chemistry, School of Pharmacy, International Medical University, Kuala Lumpur 57000, Malaysia; mallikarjunarao_pichika@imu.edu.my (M.R.P.); kitkaymak@imu.edu.my (K.-K.M.); 3 Clinical Dentistry Division, School of Dentistry, International Medical University, Kuala Lumpur 57000, Malaysia; abhishek_parolia@imu.edu.my; 4 Children and Community Oral Health, School of Dentistry, International Medical University, Kuala Lumpur 57000, Malaysia; muneer_babar@imu.edu.my

**Keywords:** arginine, biofilm, crystals, matrix metalloproteinase, Raman spectroscopy

## Abstract

This study’s objective was to examine L-arginine (L-arg) supplementation’s effect on mono-species biofilm (*Streptococcus mutans*/*Streptococcus sanguinis*) growth and underlying enamel substrates. The experimental groups were 1%, 2%, and 4% arg, and 0.9% NaCl was used as the vehicle control. Sterilised enamel blocks were subjected to 7-day treatment with test solutions and *S. mutans*/*S. sanguinis* inoculum in BHI. Post-treatment, the treated biofilms stained for live/dead bacterial cells were analysed using confocal microscopy. The enamel specimens were analysed using X-ray diffraction crystallography (XRD), Raman spectroscopy (RS), and transmission electron microscopy (TEM). The molecular interactions between arg and MMP-2/MMP-9 were determined by computational molecular docking and MMP assays. With increasing arg concentrations, bacterial survival significantly decreased (*p* < 0.05). The XRD peak intensity with 1%/2% arg was significantly higher than with 4% arg and the control (*p* < 0.05). The bands associated with the mineral phase by RS were significantly accentuated in the 1%/2% arg specimens compared to in other groups (*p* < 0.05)**.** The TEM analysis revealed that 4% arg exhibited an ill-defined shape of enamel crystals. Docking of arg molecules to MMPs appears feasible, with arg inhibiting MMP-2/MMP-9 (*p* < 0.05). L-arginine supplementation has an antimicrobial effect on mono-species biofilm. L-arginine treatment at lower (1%/2%) concentrations exhibits enamel hydroxyapatite stability, while the molecule has the potential to inhibit MMP-2/MMP-9.

## 1. Introduction

Oral biofilms are significant to both the health and disease state. The shift from homeostasis to biofilm dysbiosis is inceptive to the pathological state of chronic biofilm-mediated diseases such as dental caries [1,2]. Cariogenic biofilms are dominant with aciduric pathogens such as *Streptococcus mutans* (dysbiosis state), as their pathogenicity is dependent on frequent exposure to fermentable carbohydrates, leading to prolonged biofilm acidification and net mineral loss in dental enamel [3,4]. Indiscriminate use of anti-microbials to counter pathogenic biofilms has led to antimicrobial resistance [5,6]. The failure of anti-microbials to prevent the growth of deep-embedded pathogens through biofilms with extra-cellular polymeric substances (EPS) as a biofilm matrix constitutes the rationale for this study. Therefore, strategies are needed that combat the limitations of anti-microbials.

Biotic strategies—prebiotics, probiotics, and synbiotics—are being explored as supplementary interventions to the long-known effects of fluorides (F) on dental caries [7]. Topical F application has led to a dramatic decline in dental caries worldwide [8,9]; however, its antimicrobial effect on cariogenic biofilms is limited [10,11]. The caries-preventive potential of synbiotics is still in the pre-clinical investigation stages [7], while probiotics are effective on pathogenic biofilms [12,13,14], with brief sustainability in the oral cavity [13,14]. The role of prebiotics in caries prevention (through clinical trials) has been explored over the past two decades, with reported superior caries-preventive potential than fluoride [15,16,17,18,19,20,21,22,23,24]. Prebiotics such as arginine (arg) enhance the growth of arginolytic commensals (i.e., *Streptococcus sanguinis*, *Streptococcus parasanguinis*, *Streptococcus gordonii*, etc.) producing ammonia metabolite, which increases biofilm pH and makes the environment non-conducive to the growth of pathogens such as *S. mutans* [25,26]. Based on the promising caries-preventive potential of the prebiotic arg through clinical trials [15,16,17,18,19,20,21,22,23,24], further exploration of its mechanistic properties might aid in explaining its distinguished role in caries prevention.

The biofilm modulation potential of L-arginine has been explored through several studies. The prebiotic biofilm modifier impacts the growth of pathogens, the EPS matrix, ecological interactions, and biofilm biomass to maintain homeostasis [27,28]. At higher arg concentrations, the prebiotic inhibits the 24 h mono-species planktonic/biofilm cultures of *S. mutans* and *S. sanguinis* [29]. Conversely, with prolonged treatment (115 h), the gene expression with *gtf*B (for insoluble glucans) and SMU.150 (bacteriocin) were upregulated as opposed to the decreased expression with initial treatment [28]. Therefore, studies are needed to identify the effect of prolonged arg treatment on mono-/multi-species biofilms, given that arg is available in commercial toothpaste for daily use.

The remineralisation potential, enamel F uptake, and antimicrobial effect of F in the presence of arg were explored by our previous studies [30,31,32,33,34]. Although studies have investigated the effect of arg on the biofilms and substrates (i.e., HA discs, enamel, etc.), separately, none of the studies so far have jointly examined the effect of L-arg supplementation on the biofilms and proximate enamel substrates as the intervention is indicated for primary/secondary caries prevention. Thus, the objective of the present study was to examine the effect of L-arg supplementation on the growth of mono-species biofilms (*S. mutans*/*S. sanguinis*) and the underlying enamel substrates. In addition, the effect of L-arginine on MMPs was also evaluated. The null hypothesis tested in the present study was that there is no difference between the effect of L-arg (in vehicle 0.9% NaCl) and the vehicle control –0.9% NaCl on the mono-species biofilms (*S. mutans*/*S. sanguinis*) with the underlying substrates when subjected to once daily treatment for a 7-day treatment cycle.

## 2. Results

### 2.1. Confocal Imaging

**Figure 1:**
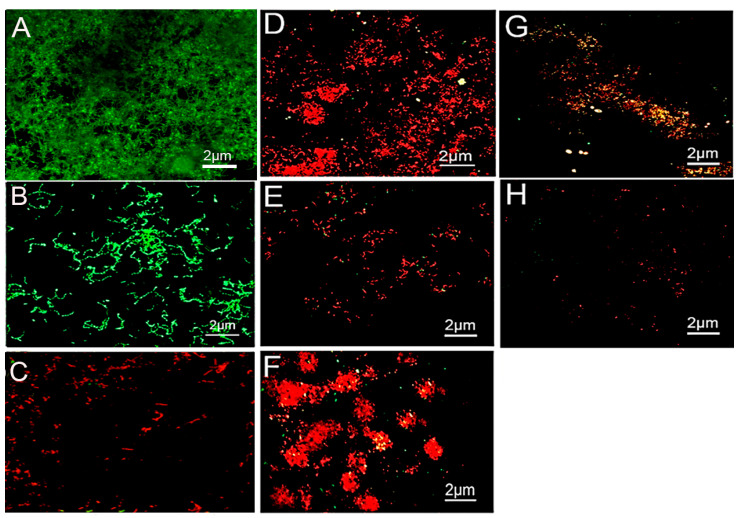
Representative CLSM images of (**A**) *S. mutans* and (**B**) *S. sanguinis* grown on enamel discs for 7-days with 0.9% NaCl treatment. (**C**) 1% arg-treated enamel specimens with *S. mutans*. (**D**) 2% arg-treated enamel specimens with *S. mutans*. (**E**) 1% arg-treated specimens with *S. sanguinis;* and (**F**) 2% arg-treated specimens with *S. sanguinis*. (**G**) 4% arg-treated *S. mutans;* and (**H**) *S. sanguinis* specimens.

The weighed Kappa for intra-observer reproducibility exceeded the 0.70 cut off, with a mean of 0.83, indicating almost perfect reproducibility for inter-observer reproducibility (Table 1). The CLSM images of *S. mutans* and *S. sanguinis* biofilms treated with the control 0.9% NaCl are shown in Figure 1A,B, respectively. The bacteria were stained green, displaying densely clustered bacterial biofilms with almost no areas of dead cells after 7 days. The 1% arg-treated specimens appeared with dead *S. mutans* bacterial colonies with few areas of surviving green-stained bacteria (Figure 1C). The 2% arg-treated enamel specimens with *S. mutans* biofilm show areas of dead and minimal vital bacterial colonies (Figure 1D), with a similar presentation of *S. sanguinis* biofilms in 1% arg (Figure 1E) and 2% arg-treated groups (Figure 1F). The biofilms treated with 4% arg also displayed completely dead microbial colonies in addition to bacterial eradication/dislodgement of cells with *S. mutans* (Figure 1G) and *S. sanguinis* (Figure 1H). Further data on the live/dead bacterial proportions are shown in Table 1.

The computed live bacterial proportions for both *S. mutans* and *S. sanguinis* with 2%/4% arg treatment were significantly lower than those for the 1% arg group, followed by the controls (*p* < 0.05). The lowest dead bacterial proportions were observed for the control (0.9% NaCl) group, followed by 1% arg treatment (*p* < 0.05).

Thus, the results of CLSM demonstrate that with increasing concentrations of arg treatment, bacterial survival in the mono-species biofilm decreased, suggestive of imparting an antimicrobial effect.

### 2.2. Hydroxyapatite Crystallographic Analysis

**Figure 2:**
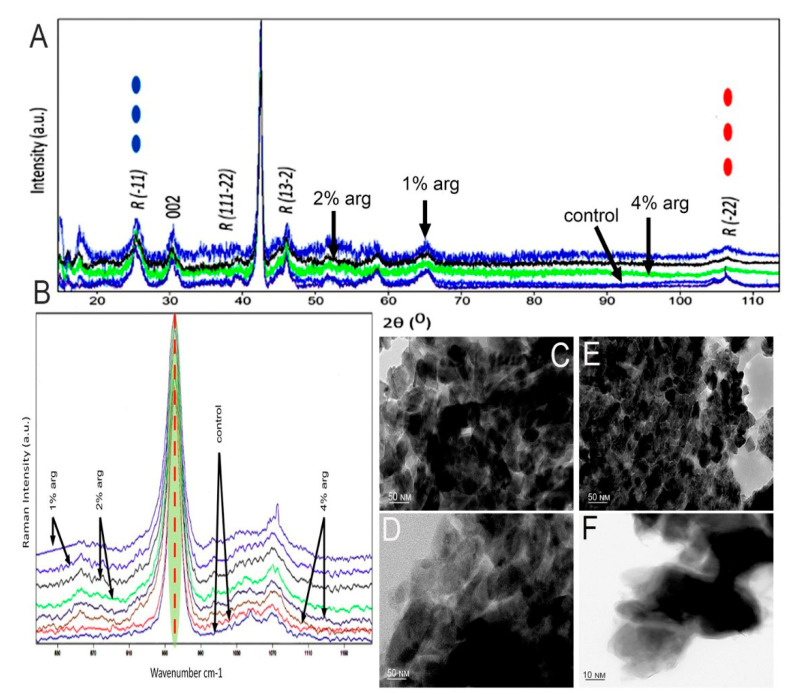
(**A**) XRD patterns of the tested experimental groups. (**B**) Representative Raman spectra recorded in the region of different experimental specimens of the hydroxyapatite band at 960 cm^−1^. Representative TEM images of (**C**) control specimen; (**D**) 1% arg-treated specimen; and (**E**) 2% arg-treated specimen. (**F**) High-magnification TEM image taken from the more electron-dense regions of the 4% arg-treated specimens. The dark spots are artefacts.

The data on the XRD analysis of the treated enamel specimens are shown in Figure 2A. The traditional approach was to understand the conformational change within the crystal structure of the enamel at a macromolecular level. As shown, the diffraction peaks enabled by 2θ at 25.8–26.0, 32.2, 48–49 and 52.9 are representative of the hydroxyapatite XRD peaks of the experimental groups. In the post-treatment cycle, the peak intensity for the 1% and 2% arg groups was significantly higher than that for the 4% arg and control group (*p* < 0.05). Reduction in the crystallite size was observed for the 1%/2% experimental groups, indicating stabilisation of the hydroxyapatite matrix (Table 2).

Raman spectroscopy was performed to understand the conformational and dynamic structure associated with the inorganic phase of the treated enamel specimens (Figure 2B). The bands associated with the mineral phase were significantly accentuated in 1% and 2% arg specimens compared to in the other groups (*p* < 0.05), corresponding to the PO-stretching identified at 960–962 cm^−1^. These bands represent symmetric phosphate stretching (A1), labelled as PO_4_^3−^ v1 (Table 2).

The representative TEM images of treated enamel specimens are shown in Figure 2C−F. The investigation focused on the morphology and the interface/shape of the crystallites. The crystallites appear mostly as elongated or hexagonal in the control (Figure 2C) specimen, demonstrating the continuous presence of HAP crystals. Figure 2D indicates the area of aggregated crystals in 1% arg specimens, compared to Figure 2E, whereby 2% arg specimens show the HAP crystallites in a perpendicular orientation to the image plane. The lattice planes are more visible with few crystallites overlapping each other and, in some cases, seem to be in contact with each other. Most of the dark spots observed are artefacts. The high-magnification TEM images from the more electron-dense regions for the 4% arg group specimens exhibit an unclear and undefined shape of the enamel crystal, as the crystallites were separated by larger gaps (Figure 2F). This may have been due to the breach of crystal boundaries in 4% arg-treated specimens.

In summary, the results of HAP crystallographic analysis suggest that with the administration of lower arg concentration (1%/2%) treatment to mono-species biofilms on enamel substrates, the enamel HAP crystals appear stable. Meanwhile, with a 7-day treatment of L-arg at 4% *w*/*v*, the HAP crystal boundaries appear disorganised, exhibiting an unusual effect on the enamel substrates.

### 2.3. Molecular Docking

**Figure 3:**
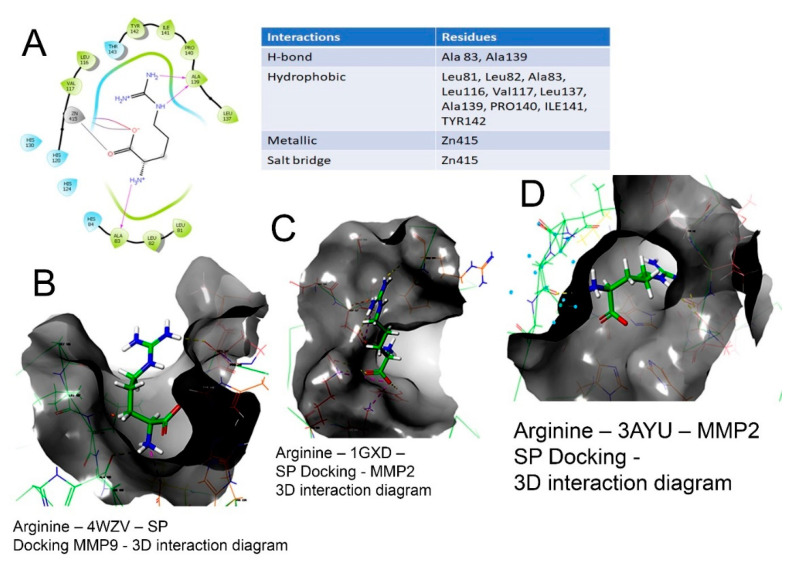
Binding of L-arg on the active site of MMPs protein as assessed by molecular docking. (**A**) Arginine–3AYU–SP docking-2D interaction diagram. The 2D surface view of docking of arg (blue) in the (**B**) catalytic cavity of MMP-9; it interacts with Zn as arg 149, forming hydrogen bonds extending in the S1′ pocket. (**C**) The docking of arginine in the MMP-2 protein shown in 3D form with the secondary structure of protein visible. (**D**) The surface view of docking of arg in the catalytic cavity of MMP-2 and the interaction of arg.

The 2D and 3D interaction of arg within the binding site, the docking scores, IFD scores, the receptor residues involved in binding and the nature of the interaction (hydrogen bonding, hydrophobic bonding, etc.) are shown in Figure 3. The detailed observations of the docking poses of arg and its interactions with key residues of the binding site in all the three docking protocols (SP, XP and Induced-Fit) revealed that the interactions are consistent with reasonable docking and IFD docking scores (Table 3). The negative scores indicate that the binding of arg within the MMP-2 and MMP-9 binding sites are favourable.

Therefore, the molecular interactions of arg to MMPs suggest that the docking of the molecule (arg) to the MMPs appears feasible with a potential to inhibit MMP-2 and MMP-9.

### 2.4. MMP-2/MMP-9 Inhibitory Activity

**Figure 4:**
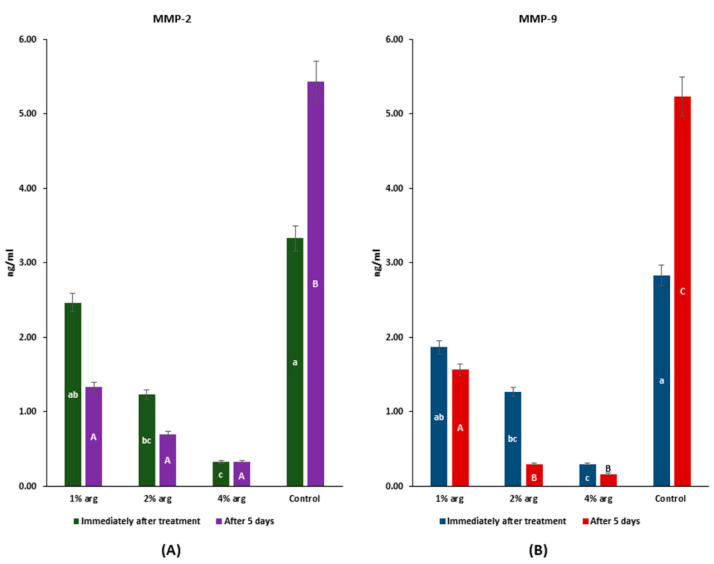
MMP assay to determine the interactions of arg with (**A**) MMP-2; and (**B**) MMP-9. The lowercase (a–c) and uppercase (A–C) letters identify significant differences between the experimental groups, immediately after the treatment and after 5 days, respectively.

The data on MMP-2/MMP-9 recognition profiles are shown in Figure 4. Immediately after treatment, the MMP-2 activity with 2% and 4% arg was significantly inhibited compared to the control (*p* < 0.05), whereas after 5 days, the MMP-2 activity with arg treatment was significantly lower than that of the control (*p* < 0.05) (Figure 4A). The MMP-9 activity was significantly inhibited with 2% and 4% arg treatment compared to the control, both immediately after treatment and with 5 days of incubation (*p* < 0.05).

The results of MMP-2/-9 activity confirm that at higher concentrations of arg (i.e., 2%/4%), the activity of MMPs is significantly inhibited (*p* < 0.05).

Overall, the results of the present study explain that arg demonstrates an antimicrobial effect on mono-species (*S. mutans*/*S. sanguinis*) biofilms. At lower concentrations (i.e., 1%/2%), arg aids in maintaining the proximate enamel HAP stability, whereas at higher concentrations (i.e., 4%), the HAP crystal mass and inter-crystal areas appear disorganised. Furthermore, arg inhibits MMP-2 and MMP-9, as the molecule could reasonably dock with the MMPs.

## 3. Discussion

Based on the experimental study design and the employed characterisations, we identified the multi-potential role of arg on the bacterial biofilms and the underlying enamel substrates. The results of the present study show that with increasing concentrations of arg, the intervention imparts an antimicrobial effect on mono-species biofilms (*S. mutans*/*S. sanguinis*). Furthermore, the results of the enamel crystallographic analysis revealed that at concentrations of 1%/2% by wt. of arg, enamel HAP crystallites demonstrate stability, suggestive of biofilm–enamel interface homeostasis. Therefore, the null hypothesis of the present study was partially rejected, as the effect of arg on enamel HAP is concentration-dependent, with higher concentrations (i.e., 4% *w*/*v*) imparting a possible deranged effect on the underlying substrate. As additional data, we also encountered that arg can reasonably dock MMPs, and thus can be a potential MMP-2 and MMP-9 inhibitor, which was further confirmed by experimental evaluations.

The bipolar semi-essential amino acid ‘arg’ is available in micromolar concentrations in the saliva. The substantial presence of salivary arg significantly increases the arginolytic bacterial arginine deiminase system (ADS) activity, thereby enhancing the growth of the commensals, leading to a homeostatic microbial consortium usually seen in caries-free individuals [25,35,36]. Prevention of dental caries is a global challenge (especially in high-risk patients), as 2.4 billion individuals worldwide are affected by untreated carious lesions [37,38]. As the effect of topically applied F in caries prevention is reaching a plateau, researchers are identifying routes to counter the limitations of F. Thus, arg appears as a promising supplemental intervention, since its substantial presence in the oral cavity is associated with the caries-related disease-free state, while demonstrating an effect on oral biofilms by utilising its prebiotic potential. Further understanding of the potential of arg to prevent caries is significant for its long-term use, as recently available oral care products contain arg and F, both.

In the present study, we discerned that with increasing concentrations of arg, a significantly higher antimicrobial activity prevails in mono-species biofilms. The results are in accordance with a previous study, whereby through a checkboard microdilution assay and CLSM, the authors of the study showed that at higher concentrations (10% by wt.), the growth of bacteria and subsequent biofilms was attenuated [29]. However, the study was performed over a short duration of 24 h, while this study was undertaken for 7-days with once-daily treatment. Thus, the results of the study emphasise that with prolonged use of arg on mono-species biofilms, an enhanced antimicrobial effect will be evident. One needs to be cautious when interpreting the results of the study, as the prebiotic effect of arg is more evident with multi-species biofilms representing the oral microbial composition. This can be explained based on the results of another study, which highlights that the growth inhibitory effect of arg was predominantly on *S. mutans* in the presence of *S. gordonii* and *A. naeslundii* [28].

To observe the effect of arg on the biofilm substrates (enamel blocks, per case), we characterised the specimens using XRD, Raman spectroscopy, and TEM. The crystallites with 1%/2% arg groups demonstrated a stabilised HAP structure (similar to control); however, with 4% arg treatment, the underlying substrates had an undefined shape with larger inter-crystal gaps. Proteins play a significant role in forming crystal mass during mineralisation. Arginine is an amino acid which forms the structural units of proteins, from which we can anticipate its potential role in remineralisation sequelae, including crystal mass changes [39,40]. However, the crystal mass stabilisation was observed with lower (i.e., 1%/2%) concentrations of arg and not with 4% arg treatment. A previous study precluded the possibility of forming an arg–phosphate complex with the treatment of incipient carious lesions by arg-F toothpaste [31]. It is quite possible that prolonged treatment with higher arg concentrations led to increased arg–phosphate complex mass, thereby affecting the existent HAP crystals’ orientation. Furthermore, it is explained that the higher concentrations of arg brings about a complex supersaturation over the crystal mass, leading to primary HAP-bounded arg, as the overall charge for the molecule (arg) is positive while the surface charge for the enamel is negative [41,42]. Meanwhile, at lower concentrations (i.e.,1%/2%), the prebiotic arg was assimilated into the biofilm. The role of higher concentrations (≥4%) of arg and the subsequent crystal mass changes can be further delineated by examining the effect of biofilm dysbiosis-resultant acid attack on the treated substrates in future studies. Furthermore, the crystals with 1% and 2% arg treatments display a slight reduction in crystal size (Table 3), indicating a stable structure and suggesting superior mechanical properties [43]. However, this needs to be further investigated.

So far, none of the studies have investigated the potential of arg to inhibit MMPs. As a first investigation, we identified that arg has the potential to inhibit MMPs using computational molecular docking and MMPs immunoassay. A previous study expressed the concern that under clinical conditions (short contact time), F alone might not be sufficiently effective in MMP inhibition, even with high-F concentration varnish [44]. As studies have shown the synergistic/enhanced effect of arg-F [45], it is quite possible that the combination can better inhibit the rate of demineralisation of dentin lesions. As arg is a bipolar amino acid, the negative charge terminal (although not dominant) might interact with positively charged Ca/Zn to prevent the catalytic process. Furthermore, the dominant positive charge of arg is free to interact when supplemented with F, while F can append interaction with the Ca/Zn ions of MMPs. Additionally, considering the limitations of F as a dentin-targeted anti-erosive agent [46] and, concurrently, the acid dissociation constant (as arg pKa is 12.48) of arg, it appears that the combination will improve the anti-erosive properties of F. Arginine is an antagonist of nitric oxide (NO) synthase [47], meaning that less NO will be produced in its presence. In an in vitro or in vivo context, this inhibition of NO may reduce the cleavage of MMP. This potency of MMP inhibition was further elucidated by the molecular docking simulation of arginine over MMPs. However, further investigations are needed to explore the proposed mechanisms of arg with/without F for its action to prevent dentin lesion demineralisation.

Exploring multiple possibilities where arg can impart its eminent effect, we can now estimate that arg has a concentration-dependent effect on biofilms, enamel, and dentin. With immense biotherapeutic potential, vehicles through which the proposed arg interventions can be dispensed are yet to be ascertained. With a comprehensive study performed, we note the limitations for future studies. As arg is known to impart its effect on ecological interactions between the pathogens and commensals to effectuate homeostasis, the present study limited to examining the effect of arg treatment on mono-species biofilms of *S. mutans* and *S. sanguinis*. However, the present study explains the multi-potential role of arg on mono-species biofilm through the characterisation of biofilms treated on enamel substrates using methods such as Raman spectroscopy and TEM, which is novel to the reported studies on arg. As the effect of the intervention (arg) is now explored through these characterisations, further studies (utilising these characterisations) are needed to explain the effect of arg treatment on multi-species biofilms grown on enamel substrates. Apart, the current model utilised for the treatment cycle is a static closed model that does not account for somatic parameters existent with life. Hence, robust open biofilm models are needed to further explore the effect of the intervention with arg that can simulate body parameters while representing the oral microbial consortia.

## 4. Materials and Methods

### 4.1. Experimental Study Design

**Figure 5:**
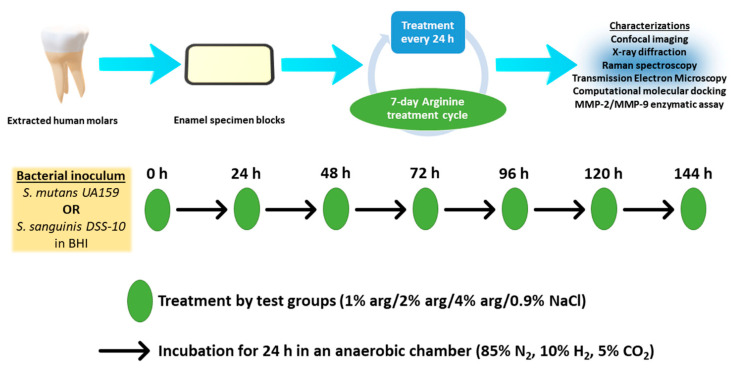
Experimental study design.

The present study’s experimental design is depicted in Figure 5. Briefly, enamel specimens and treatment solutions were prepared. The enamel specimens were sterilised by placing specimens in autoclave paper sealed with autoclave tape and were subjected to steam autoclaving (Series 32 Model DS38, Rodwell Scientific Instruments, Hornchurch, Essex, England) at 121 °C for 5 min followed by 10 min of air drying below atmospheric pressure. The specimens were than subjected to the growth of mono-species biofilms with *S. mutans* and *S. sanguinis*. The treatment solutions were co-delivered with the prepared bacterial inoculum on the enamel substrates during the onset of the 7-day treatment cycle. In the post-treatment cycle, the enamel specimens with biofilms were characterised with confocal laser scanning microscopy (CLSM), X-ray diffraction crystallography (XRD), Raman spectroscopy, and transmission electron microscopy (TEM). As an additional investigation, we explored the possibility of arg-MMP interactions using computational molecular docking and MMP assay.

### 4.2. Enamel Specimen Preparation

Sound human third molars (*n* = 80) were stored in 0.2% sodium azide at 4 °C to inhibit bacterial growth, and the teeth were used within one month post-extraction. A priori, the experimental study protocol was approved by the Institutional Review Board of International Medical University Kuala Lumpur (grant number #492/2020).

The enamel specimens (3 × 3 × 2 mm^3^) were prepared using ISOMET Low-Speed Saw (Isomet, Buehler, Lake Bluff, IL, USA) from the buccal surface with two diamond discs (Extec Corporation, XL-12205, Enfield, CT, USA). The specimens were standardised for surface roughness using the Mitutoyo Surface Roughness Tester (SJ-210) indicated by Ra value. After preparation, the specimens were placed in an ultrasonic bath (T7 Thornton, Unique Ind. e Com. Ltd.a., São Paulo, Brazil) for cleaning for 15 min.

### 4.3. Treatment Solutions

The arg treatment solutions were prepared in the vehicle, 0.9% NaCl, that served as a control. L-arginine (A-5006, Sigma Aldrich, St. Louis, MO, USA) were weighed at 100, 200, and 400 mg to be suspended in a 10 mL of 0.9% NaCl. The suspension was vortexed until the arg solute was completely solubilised. Fresh treatment solutions were prepared each time the substrates were scheduled to receive treatment.

The treatment groups in the present study were–

Group 1: 100 mg arg in 0.9% NaCl (1% arg)

Group 2: 200 mg arg in 0.9% NaCl (2% arg)

Group 3: 400 mg arg in 0.9% NaCl (4% arg)

Group 4: 0.9% NaCl (control)

### 4.4. Bacterial Inoculum and Experimental Cycle

The commercially obtained bacteria from American Type Culture Collection (ATCC), *S. mutans* UA159 (ATCC 700610) and *S. sanguinis* DSS-10 (ATCC 10556), were routinely grown for 3 days in BHI under anaerobic conditions (85% N_2_, 10% H_2_, 5% CO_2_, 37 °C). To prepare the bacterial inoculum, after routine washing three times (using sterile phosphate-buffered solution, 0.01 M, pH 7.2) and centrifugation (4000 rpm for 10 min), the bacterial cells were adjusted to a concentration of 10^7^ cells/mL in BHI broth. Each of the cell pellets was washed thrice with sterile phosphate buffered solution (PBS, 0.01 M, pH 7.2). The turbidities of the bacterial cell suspensions were measured using Bausch and Lomb Spectronic 88 spectrophotometer (Milton Roy Spectronic, Rochester, NY, USA) by measuring the optical density at 600 nm (OD_600 nm_) to avoid strong UV absorbances of dissolved molecules, as the defined wavelength helped to standardise the measurements. Each suspension was adjusted to 75% ± 1% transmission. The BHI broth was supplemented using 8% sucrose (D(+)-saccharose; Sigma-Aldrich Korea (Seoul, Korea) with a stated purity of >99 wt%) (pH 7.4) [48] and a minimal amount of xylitol (0–2%) at 37 °C for 48 h. The solution was sterilised by filtering with 0.2 µm filters followed by incubating for a week, at room temperature to ensure sterility.

The sterilised enamel specimen blocks were introduced in a microplate and subjected to inoculum co-cultured with the equal volume of NaCl in treatment solutions. The microplate with substrates, bacteria, and treatment solutions was incubated in an anaerobic chamber (85% N_2_, 10% H_2_, 5% CO_2_) for 24 h. After each 24 h, the spent BHI media was withdrawn to furnish the substrates with biofilms to fresh BHI media and arg/control treatment. The 24 h treatment cycle was continuously performed for seven days. The substrates with biofilms were then removed and dip-washed in PBS followed by characterisations. Biofilm was removed with a Satelec P5 Newtron ultrasonic generator with the tip 10P (Satelec, France) held 0.5 or 1 mm away from disc; the scaler was operated for 30 or 60 s. The procedure was performed under room temperature.

### 4.5. Confocal Laser Scanning Microscopy

The bacterial vitality of mono-species biofilms was examined using CLSM (Fluoview FV 1000, Olympus, Tokyo, Japan) and the Live/Dead Baclight bacterial viability kit (molecular probe #L7012 LIVE/DEAD BacLight stain; Invitrogen) as per the manufacturer’s instructions. The specimens with biofilms were stained with the viability kit and incubated at room temperature for 30 min, prior to confocal laser scanning. Precautions were taken to avoid photo-interference of the stained specimens, and thus the microplate was thoroughly covered with aluminium foil. The excess stain was eradicated from specimens using absorbent paper and biofilms were imaged on a CLSM using light emission between 500 and 550 nm with an excitation wavelength of 488 nm at 100× objective lens for direct observation at five randomly chosen view fields (25 µm depth). Bioimage L software (v.2.0. BioImage XD, Malmő, Sweden) was used to analyse confocal images on a two-dimensional x-y section, based on colour segmentation algorithms written in MATLAB. The respective proportions for live/dead bacteria for each treatment group were calculated using Image J software (v. 1.53e, NIH, Bethesda, Maryland, MD, USA) with a new binarisation approach considering cocci, which form clusters. The binarised images obtained after thresholding were then processed by watershed segmentation, to separate touching cells, followed by an automated edge detection algorithm. In this way, we were able to count the percentage of cells considering the parameters cell area and perimeter. To determine inter-observer reliability for the CLSM images, selected images from groups were randomly chosen, and the second and blinded investigator repeated the manual analysis.

### 4.6. X-ray Diffraction Crystallography

In the post-treatment cycle, the enamel specimens were subjected to XRD analysis. The specimens were step scanned from 15° to 70° of 2θ and a step size of 0.02° using a Bruker D8 Advance X-ray diffractometer (–goniometer) (Bruker Corporation, Billerica, MA, USA), having a goniometer radius of 217.5 mm at 40 kV. The opening angle was set at 3.5°. The secondary optics were performed using an 8 mm antiscatter slit and a 2.5° Soller slit. The patterns were evaluated in TOPAS software (TOPAS 4.2, 2009, Bruker AXS GmbH; Karlsruhe, Germany) and crystal sleuth with Gaussian functions for the crystallisation effect at β_LS_. The peak broadening was discarded to avoid the micro strain effect of the biological origin of samples. The crystallite size was evaluated using the integral formula of:

βi = λ/ξ. cos θ(1)

All experiments were conducted in triplicate to ensure sufficient reproducibility.

### 4.7. Raman Spectroscopy

Raman spectroscopy was carried out on enamel specimens using a JY LabRam HR 800 Raman spectrometer (Horiba Jobin Yvon, Longjumeau, France) equipped with Leica lenses and curve-fitting Raman microscopic software (Labspec 5). A 40× objective with 785 nm of a near infra-red laser was used to collect Raman spectrum at a power of approximately 20 mW with the integration of 10 s each. The spatial resolution of 5 μm was set with the central and outside regions for all focused specimens. Normal enamel intensities were also measured in positive controls (unmodified enamel cuts) with a 4th-order polynomial fit fixed at 930–960 cm^−1^ to remove fluorescence spectra, identifying the mineral component. All experiments were conducted in triplicate to ensure sufficient reproducibility.

### 4.8. Transmission Electron Microscopy

The enamel specimen crystal orientation of experimental groups (*n* = 3) was analysed using TEM. The TEM micrographs (JEOL-1010, Joel Jem, Tokyo, Japan) were obtained using an operating voltage of 200 kV. Vertical sections were made in an occlusal apical direction for each enamel specimen block to separate the enamel. The enamel specimen slabs were fixed, buffered using 0.1 M sodium cacodylate for 1 h, and rinsed with distilled water. Then, the specimens were dehydrated using ascending concentrations of ethanol and finally infiltrated with araldite resin. Ultrathin sections (~90 nm) were prepared using ultra-microtome and a diamond knife with specimens collected on grids. The specimens were eventually stained using uranyl-acetate, UO_2_(CH_3_COO)_2_·2.H_2_O, for 10 min. The samples were prepared by dispersing the slabs in ethanol under ultrasonication. The sample was then placed on the copper grid and coated with carbon film for analysis of hydroxyapatite (HAP) with TEM.

### 4.9. Molecular Docking

As an additional objective of this study, we determined the molecular interactions between arg and the proteins MMP-2 and MMP-9 using computational molecular docking. The molecular docking was performed on crystal structures; 3AYU (MMP-2 with zinc binding site), 1GXD (MMP-2 with non-zinc binding site), 4WZV (MMP-9 conformation A with zinc binding site), 4H2E (MMP-9 conformation B with zinc binding site) and 5TH9 (MMP-2 with non-zinc binding site). Glide standard precision (SP), extra precision (XP) mode and induced-fit docking modules of Schrodinger 2020–2 were used to determine the interactions between arg and proteins. From the binding energy and interaction studies, arg projected molecular interactions with both MMP-2 and MMP-9.

#### 4.9.1. Proteins Preparation

The crystal structures of MMP-2 (3AYU, and 1GXD) and MMP-9 (4WZV, 4H2E, and 5TH9) were downloaded from https://www.rcsb.org/ on 12 November 2020. The proteins were prepared for molecular docking studies using the protein preparation wizard using OPLS-3e force field at pH 7.0 ± 0.20 and the other default settings. As the resting pH of the oral cavity ranges from 6.80 to 7.20, the protein was prepared at a neutral pH 7.0, with a deviation of ±0.20.

#### 4.9.2. Binding Site Detection

The zinc or S1 binding pocket of MMP-2 (PDB ID: 3AYU) was defined using the amino acid residues (140–150 of Ω loop sequence) as the centroid of the cavity and the receptor binding site was generated using the receptor grid generation module with OPLS-3e force field and default settings. The non-zinc binding site of MMP-2 (PDB ID: 1GXD) was defined using Phe621 as the centroid of the cavity, and the receptor binding site was generated using the receptor grid generation module with OPLS-3e force field and default settings.

The zinc or S1 binding pocket of MMP-9 in A conformation (PDB ID: 4WZV) was defined using amino acid residues (246–256 of Ω loop sequence) as the centroid of the cavity and the receptor binding site was generated using the receptor grid generation module with OPLS-3e force field and default settings. The zinc or S1 binding pocket of MMP-9 in B conformation (PDB ID: 4H2E) was defined using amino acid residues (246–256 of Ω loop sequence) as the centroid of the cavity and the receptor binding site was generated using the receptor grid generation module with OPLS-3e force field and default settings. The non-zinc binding site of MMP-9 (PDB ID: 5TH9) was defined using arg162 as the centroid of the cavity, and the receptor binding site was generated using the receptor grid generation module with OPLS-3e force field and default settings.

#### 4.9.3. Arginine Docking

Glide module was used to dock the arg molecule into the binding site grid using standard precision (SP) and extra precision (XP) docking protocols with default settings. In SP and XP docking protocols, the ligand was made flexible, and the receptor was made rigid. To further confirm the binding efficacy of arg, induced-fit docking studies were performed using the ‘Induced-Fit docking’ module. In this, the ligand was made flexible, and all the residues within the range of 5 Å of the receptor were made flexible. In general, the induced-fit docking provides better insights on binding interactions efficacy.

### 4.10. MMP-2 and MMP-9 Assay

To verify the MMP-2/-9 inhibitory activity, an assay was performed using human recombinant active MMP-2 (Calbiochem, La Jolla, CA, USA) and human neutrophil MMP-9 (Calbiochem, La Jolla, CA, USA), as per the manufacturer’s instructions. Purified standards were prepared by diluting the commercial buffers with the addition of 5 μg/μL of BSA (Sigma, St. Louis MO USA). Then, the calibration curve to protease concentrations was determined. The dialysed supernatants were incubated with their respective buffers (10 mM CaCl, 50 mM Tris, 150 mM NaCl) at 37 °C for 1.5 h. After dilution of activated MMPs, 80 μL solutions were transferred into a 96-well microplate. The treatment solutions (10 µL) were added into the corresponding wells for 5 min at room temperature (27 °C), and end-point absorbance was measured (after adding detection reagents) using a spectrophotometer at OD_405nm_ (Bio-Rad Laboratories, Inc. Hercules, CA, USA). The protease concentrations were expressed as ng/mL immediately after treatment and after 5 days of incubation. All experiments were conducted in triplicate to ensure sufficient reproducibility.

### 4.11. Statistical Analysis

The data were entered in an MS Excel sheet (Microsoft Office Professional Plus 2019, Microsoft, Redmond, WA, USA) and subjected to statistical analysis using SPSS v. 26 (IBM statistics, New York, NY, USA). The proportional data on live/dead bacterial cells and MMP-2/MMP-9 activity data were analysed using 1-way ANOVA with Tukey’s HSD post hoc test. For nanoleakage evaluation, the inter-observer reproducibility and the intraobserver reproducibility were evaluated using the weighted Kappa (kw) statistic.

The statistical significance was set at α = 0.05.

## 5. Conclusions

Under the conditions of the present study, we conclude that:

1. Increasing concentrations of arg impart an antimicrobial effect on mono-species biofilm with long-term treatment.
2. Sustained 1%/2% L-arg treatment maintains a stable enamel hydroxyapatite crystal, demonstrating biofilm–enamel interface homeostasis.
3. L-arginine has the potential to inhibit MMP-2 and MMP-9 with reasonable docking.

## Figures and Tables

**Table 1:** Bacterial live/dead proportions in mono-species biofilms treated for 7-days.

Groups	Live (%)–Mean (SD)	Dead (%)–Mean (SD)	Calculation of Percent Difference
***S. mutans***			
Control	97.1 (10.1) ^a^	2.9 (0.7) ^1^	0
1% arg	34.4 (8.1) ^b^	65.6 (5.4) ^2^	0
2% arg	10.8 (12.4) ^c^	89.2 (6.9) ^3^	1
4% arg	5.9 (7.7) ^c^	94.1 (8.7) ^3^	−1
***S. sanguinis***			
Control	93.7 (9.1) ^A^	6.3 (1.4) ^α^	0
1% arg	40.9 (7.9) ^B^	59.1 (11.1) ^β^	0
2% arg	21.8 (5.5) ^C^	78.2 (6.1) ^γ^	0
4% arg	14.3 (4.5) ^C^	85.7 (9.9) ^γ^	1
Different superscript lowercase (a, b, c)/uppercase (A, B, C) English letters, numbers (1, 2, 3), and Greek letters (α, β, γ) represent significant differences between different treatment groups. One-way ANOVA with Tukey’s HSD post hoc test; *p* < 0.05 is significant.			Mean 0.83 *p* < 0.05

**Table 2:** Enamel Raman phosphate peaks and XRD analysis for crystallite size (in nm) with mono-species biofilms treated for 7 days.

Groups	Raman Phosphate Peaks	Crystallite Size (nm)
***S. mutans***		XRD Analysis
Control	958 cm^−1^	24.31
1% arg	960 cm^−1^	22.3
2% arg	960 cm^−1^	21.7
4% arg	964 cm^−1^	23.9
***S. sanguinis***		
Control	956 cm^−1^	
1% arg	960 cm^−1^	
2% arg	960 cm^−1^	
4% arg	963 cm^−1^	

**Table 3:** Detailed observations of docking poses of arg and its interactions with key residues of binding site in all the three docking protocols (SP, XP and Induced-Fit) with MMP-2 and MMP-9 and IFD docking scores.

Interactions	Residues
Arginine–3AYU–SP Docking (MMP2)	
H-bond	Ala 83, Ala139
Hydrophobic	Leu81, Leu82, Ala83, Leu116, Val117, Leu137, Ala139, PRO140, ILE141, TYR142
Metallic	Zn415
Salt bridge	Zn415
Arginine–3AYU–XP Docking (MMP2)	
H-bond	Ala139, PRO140
Hydrophobic	Leu82, Leu116, Val117, Leu137, Ala139, PRO140, ILE141, TYR142
Metallic	Zn415
Salt bridge	Zn415
Arginine–4WZV–SP Docking (MMP9)	
H-bond	Ala189, Pro246
Hydrophobic	Leu187, Leu188, Ala189, VAl223, Pro246, Met247
Metallic	Zn302, Glu227
Salt bridge	Zn302

## Data Availability

The data will be available with the study authors.

## Sample Availability

Samples of the compounds are not available from the authors.
